# Supplementary material for: Chemical and structural heterogeneity of olive leaves and their trichomes
Source: Commun Biol. 2024 Mar 22;7:352. doi: 10.1038/s42003-024-06053-4 (PMC10960044; doi:10.1038/s42003-024-06053-4)
Supplement: Supplementary file 2 — Supplemental material [file 42003_2024_6053_MOESM2_ESM.pdf]

SUPPORTING INFORMATION

Table S1. Pseudo-equilibrium contact angles for drops of water (ϑ_w), glycerol (ϑ_{gly}) and diiodomethane (ϑ_{dm}) with the upper and lower side of young (2 to 3-months old) and old (> 1-year old) olive leaves. Data are means ± SD (N=40). For the same leaf side, different letters indicate significant differences according to Tukey´s HDS test ($P \leq 0.05$).

		Contact Angles (°)		
**Age**	**Leaf side**	Water (ϑ_ω)	Glycerol (ϑ_{gly})	Diiodomethane (ϑ_{dm})
Young	Upper	67 ± 8 a	72 ± 8 a	61 ± 5 a
	Lower	101 ± 9 a	96 ± 4 a	62 ± 5 a
Old	Upper	66 ± 8 a	64 ± 10 a	56 ± 4 a
	Lower	81 ± 8 b	91 ± 6 a	60 ± 5 a

Table S2. Surface energy and related parameters of young and old olive leaf surfaces. Total surface free energy (γ). Lifshitz van der Waals component (γ^{LW}), Acid-base component (γ^{AB}) with the contribution of electron donor (γ^{-}) and electron acceptor (γ^{+}) interactions, total surface free energy (γ_s) and polarity (% γ^{AB} γ^{-1}) of adaxial and abaxial leaf surfaces of young (2 to 3-months old) and old (> 1-year old) olive leaves.

Age	Leaf side	γ^{LW} (mJ m^{-2})	γ^{AB} (mJ m^{-2})	γ_s (mJ m^{-2})	*Polarity (%)*	δ *(MJ$^{1/2}$ m$^{-3/2}$)*
Young	Adaxial	22	8	29	26	16
Old	Adaxial	25	2	27	8	15
Young	Abaxial	25	7	32	23	17
Old	Abaxial	21	37	58	63	26

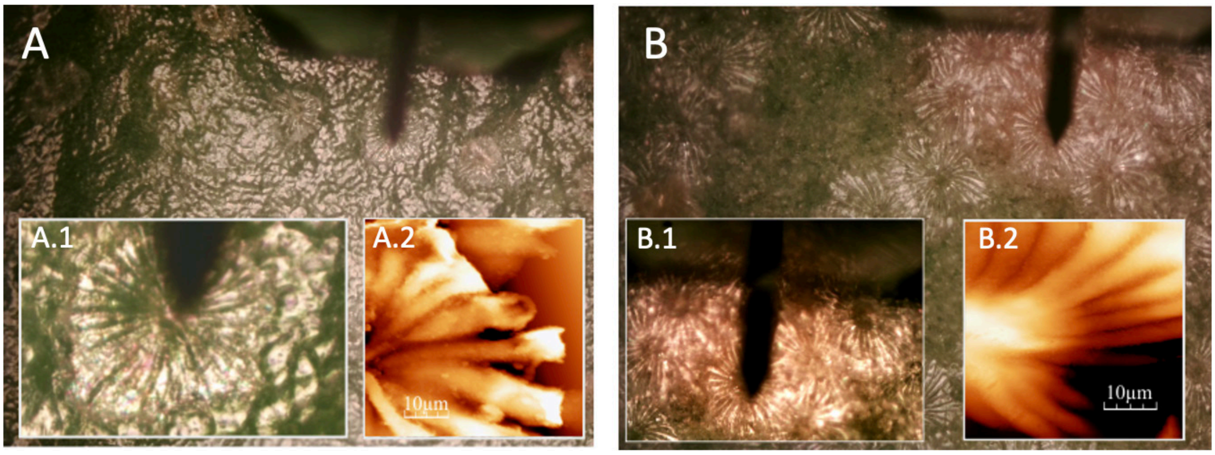

Figure S1. Optical and AFM images of the adaxial (A) and abaxial (B) sides of an olive leaf. Larger optical images show the region around the AFM cantilever (darker rectangular structure at the upper left corner of each image). The full scale of large optical images (A and B) is about 1.2x0.8mm. The smaller insets on the lower left corner of each set of images (A.1 and B.1) show OM images of trichomes, as well as the very end of the cantilever with the tip used for AFM measurement. Right insets in the lower corner of large magnification (A.2 and B.2), gold color figures are large scale AFM images of about 50 μm x 50 μm lateral size, the full color scale representing 5 μm for the left AFM image (Adaxial leaf side) and 6 μm for the right AFM image (Abaxial leaf side).

Figure S1 shows large-scale images of the adaxial (A) and abaxial (B) side of an olive leaf. The enlarged optical images (Figure S1 A.1 and B.1) prove that the tip imaging the surface is indeed located over a trichome. The AFM images (golden color scale) show the morphology of the adaxial (Figure S1 A.2) and abaxial (Figure S1 B.2) leaf side. In analogy to the OM pictures, AFM images show elongated structures (i.e., the cells composing these multicellular peltate hairs) stretching out from the center of the trichomes. Note, however, that the information given by each kind of images is fundamentally different: while OM images are related to the reflectivity of the leaf surface, AFM images show its precise morphology.

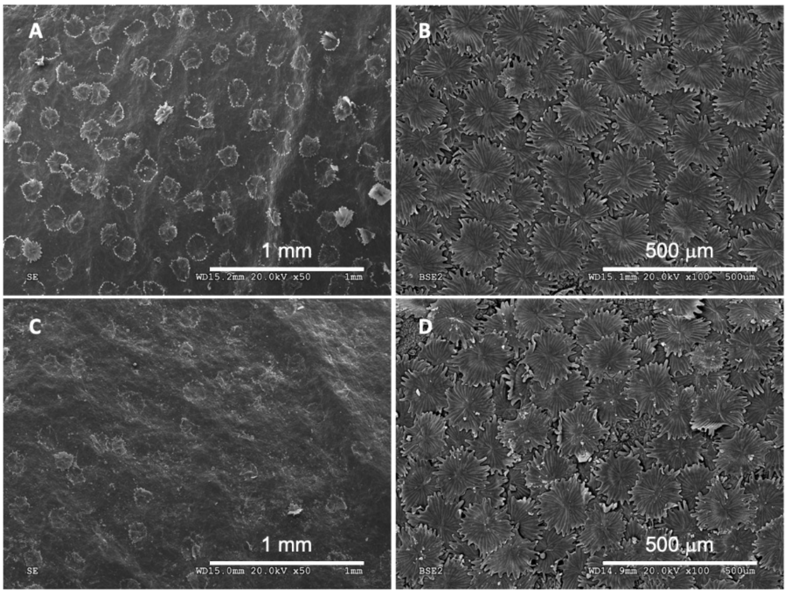

Figure S2. SEM images of adaxial and abaxial surfaces of young and old olive leaves
Scanning electron microscopy images of adaxial (**A, C**) and abaxial (B, D) surfaces of young (2 to 3-months old) and old (>1-year old) olive leaves. Young leaves have initially many trichomes in the upper side (**A**) which are either shed, degenerate or become buried in the surrounding epicuticular waxes when they become older (**C**). Lower leaf side trichomes appear intact when leaves are young (**B**) and seem to lose structure with age (**D**)
